# Supplementary material for: Hybrid Models and Biological Model Reduction with PyDSTool
Source: PLoS Comput Biol. 2012 Aug 9;8(8):e1002628. doi: 10.1371/journal.pcbi.1002628 (PMC3415397; doi:10.1371/journal.pcbi.1002628)
Supplement: Text S4 — Complete source code for the PyDSTool package (version 0.88.120504). Includes API documentation and help files linking to web pages. This file is identical to the current public release on Sourceforge.net. (ZIP) [file pcbi.1002628.s004.zip › PyDSTool/html/PyDSTool.Toolbox.ModelEst-pysrc.html]

xml version="1.0" encoding="ascii"?


PyDSTool.Toolbox.ModelEst


| Home | Trees | Indices | Help | PyDSTool |
| --- | --- | --- | --- | --- |

Package PyDSTool ::
Package Toolbox ::
Module ModelEst

[hide private]

[frames] | no frames]

# Source Code for Module PyDSTool.Toolbox.ModelEst

```
 1  """Model estimation classes for ODEs. 
 2   
 3     Robert Clewley. 
 4  """ 
 5   
 6  # PyDSTool imports 
 7  from PyDSTool.Points import Point, Pointset 
 8  from PyDSTool.Model import Model 
 9  from PyDSTool.ModelSpec import * 
10  from PyDSTool.common import Utility, _seq_types, metric, args 
11  from PyDSTool.utils import intersect, remain, filteredDict 
12  from PyDSTool.errors import * 
13  from PyDSTool.Toolbox.ParamEst import * 
14   
15  # Other imports from scipy import isfinite, mean 
16  from scipy.linalg import norm, eig, eigvals, svd, svdvals 
17   
18  from numpy import linspace, array, arange, zeros, sum, power, \ 
19       swapaxes, asarray, ones, alltrue, concatenate, rank, ravel, argmax 
20  import math, types 
21  from copy import copy, deepcopy 
22   
23  # ---------------------------------------------------------------------- 
24   
25  # !! In development 


26 -class ModelEst(Utility):


27      """General-purpose model estimation class. 
28      """ 
29      _needKeys = ['manager', 'context'] 
30      _optionalKeys = ['libraries', 'verboselevel'] 
31   


32 -    def __init__(self, **kw):


33          # MProject model manager object 
34          self.manager = kw['manager'] 
35          # model library object 
36          if 'libraries' in kw: 
37              self.libraries = kw['libraries'] 
38          else: 
39              self.libraries = None 
40          # MProject.context class 
41          self.context = kw['context'] 
42          if 'verboselevel' in kw: 
43              self.verboselevel = kw['verboselevel'] 
44          else: 
45              self.verboselevel = 1

46   


47 -    def fit(self, name, free_pars, parest=None):


48          """Fit an individual candidate model in its context, 
49          peforming parameter optimization on the given free parameters. 
50          """ 
51          model = self.manager[name] 
52          assert remain(free_pars, model.pars) == [], "Invalid free variables" 
53          model.set(verboselevel=self.verboselevel) 
54          # get initial results of context at default parameters 
55          # if success then done already 
56          if self.context.evaluate(model): 
57              return filteredDict(model.pars, free_pars) 
58   
59          # determine parameter sensitivity of the free parameters 
60          # dict of par -> sensitivity 
61          #sens = param_sensitivity(model, self.context, free_pars) 
62   
63          print "Get pre-defined tols from features somehow" 
64          print "Establish parameter estimation class and run it"

65          #pest = LMpest(freeParams=free_pars, 
66          #              testModel=model, 
67          #              context=self.context, 
68          #              verbose_level=self.verboselevel) 
69          #return pest.run(parDict={'ftol': 1e-5, 
70          #                        'xtol': 1e-5, 
71          #                        'args': (self.context,)}, 
72          #                verbose=self.verboselevel>0) 
73
```

  


| Home | Trees | Indices | Help | PyDSTool |
| --- | --- | --- | --- | --- |

|  |  |
| --- | --- |
| Generated by Epydoc 3.0.1 on Fri May 4 15:24:21 2012 | http://epydoc.sourceforge.net |
